# Post-earthquake traumatic stress and future time perspective: the indirect roles of problematic social media use and doomscrolling

**DOI:** 10.1186/s40359-026-04651-x

**Published:** 2026-04-29

**Authors:** Enver Ulaş, Harun İsmail İncekara

**Affiliations:** 1 Independent Researcher, Istanbul, Türkiye; 2 Avrasya University, Trabzon, Türkiye

**Keywords:** Post-earthquake traumatic stress, Problematic social media use, Doomscrolling, Future time perspective, Uncertainty reduction theory, Indirect effects

## Abstract

**Objective:**

Natural disasters such as earthquakes disrupt individuals’ sense of safety and temporal continuity. In response to uncertainty, individuals may increasingly engage with digital environments, which may evolve into maladaptive patterns of use. Drawing on Uncertainty Reduction Theory, this study examined whether problematic social media use and doomscrolling function as indirect pathways linking post-earthquake traumatic stress to future time perspective.

**Methods:**

A total of 414 undergraduate students from a public university in Kahramanmaraş, Türkiye, participated in this cross-sectional study. Data were collected using validated self-report measures. The hypothesized indirect effects model was tested using path analysis with bootstrapping (5,000 resamples).

**Results:**

Post-earthquake traumatic stress was positively associated with problematic social media use and limited future time perspective. Problematic social media use significantly predicted both doomscrolling and limited future time perspective. Doomscrolling did not emerge as a significant predictor of future time perspective. Indirect effects analyses indicated that problematic social media use accounted for the relationship between traumatic stress and future time perspective, whereas the indirect pathway through doomscrolling was not significant.

**Conclusions:**

The findings suggest that problematic social media use may represent a key behavioral pathway linking post-earthquake traumatic stress to individuals’ perceptions of the future. Although doomscrolling is associated with problematic social media use, it does not independently contribute to future time perspective. These results highlight the importance of distinguishing between different forms of digital engagement in understanding psychological responses to trauma.

## Introduction

Natural disasters such as earthquakes represent highly disruptive and uncontrollable events that can profoundly affect individuals’ psychological functioning. Rather than referring to trauma solely as an event, it is important to distinguish between exposure to a disaster and its psychological consequences. In this context, the present study adopts the concept of post-earthquake traumatic stress, referring to the emotional, cognitive, and behavioral responses that emerge following exposure to a disaster [1, 2]. Such experiences often involve heightened uncertainty, perceived loss of control, and disruptions in meaning-making processes, all of which may have enduring implications for psychological functioning [3–6].

In the aftermath of trauma, individuals frequently engage in various coping strategies to manage distress and restore a sense of predictability. One increasingly prominent pathway involves engagement with digital environments. Online platforms, particularly social media, provide immediate access to information, social connection, and emotional support, making them highly salient tools for coping under conditions of uncertainty [7–11]. However, while initial engagement may serve adaptive functions, prolonged and excessive use may shift toward maladaptive patterns characterized by diminished control and compulsive engagement [12–15].

Problematic patterns of social media use have been conceptualized as reflecting difficulties in self-regulation, behavioral control, and emotional regulation, rather than constituting a formal clinical addiction [12, 16]. Within this framework, problematic social media use represents a broader behavioral tendency that may increase individuals’ susceptibility to more specific maladaptive digital behaviors. Importantly, this distinction allows for a more precise conceptualization of how digital engagement may evolve from general overuse into more narrowly defined behaviors, such as repetitive exposure to negative content.

One such behavior is doomscrolling, defined as the persistent consumption of distressing or negative online information [14, 17, 18]. Doomscrolling has been linked to heightened anxiety, threat sensitivity, and emotional dysregulation, particularly in contexts characterized by uncertainty and perceived danger [19–22]. From a psychological perspective, doomscrolling can be conceptualized as a repetitive information-seeking behavior driven by heightened uncertainty and reduced tolerance for ambiguity. Rather than reflecting hypervigilance in a clinical sense, such behavior may emerge as individuals attempt to monitor potential threats and regain a sense of predictability. However, this process may become self-reinforcing, as continuous exposure to negative information perpetuates uncertainty and emotional distress rather than resolving it [17, 23]. In this sense, doomscrolling may reflect a maladaptive extension of uncertainty reduction efforts, rather than an adaptive coping strategy.

Beyond immediate emotional consequences, these digital behaviors may also influence how individuals perceive and organize their future. Future time perspective (FTP) reflects individuals’ perceptions of their remaining time, opportunities, and limitations, and plays a central role in motivation, goal-setting, and psychological well-being [24–26]. According to socioemotional selectivity theory, individuals’ perceptions of time are dynamic and sensitive to contextual factors, including stress and uncertainty [25]. Post-earthquake traumatic stress may contribute to a “foreshortened future,” characterized by reduced expectations, diminished hope, and a narrowed temporal horizon [27–29]. In this sense, both trauma-related processes and maladaptive digital behaviors may converge in shaping how individuals construe their future.

To conceptualize these processes, Uncertainty Reduction Theory (URT) provides a useful framework [30]. The theory posits that individuals are motivated to seek information in order to reduce uncertainty, particularly under conditions of ambiguity or threat [31–33]. However, excessive information-seeking may also give rise to maladaptive outcomes, including problematic social media use and doomscrolling [34–36]. Thus, digital behaviors may simultaneously function as coping mechanisms and sources of additional psychological strain.

Despite the growing body of research on trauma and digital behavior, the sequential relationships among post-earthquake traumatic stress, problematic social media use, and doomscrolling in shaping future time perspective remain insufficiently understood. The ordering of variables in the proposed model is grounded in the distinction between general patterns of digital engagement and more specific content-driven behaviors. Problematic social media use reflects a broader tendency characterized by difficulties in self-regulation, loss of control, and persistent engagement across contexts. In contrast, doomscrolling represents a more situational and content-specific behavior, defined by repetitive exposure to negative information.

From this perspective, individuals who exhibit higher levels of problematic social media use may be more likely to engage in specific maladaptive behaviors such as doomscrolling, as their general pattern of use increases both exposure to and persistence in negative content consumption. This distinction is consistent with prior conceptualizations that position problematic use as a higher-order behavioral tendency encompassing more specific digital behaviors. Although alternative temporal orderings are theoretically plausible, the present model prioritizes this direction based on theoretical considerations.

Importantly, given the cross-sectional nature of the data, this ordering should be interpreted as theoretical rather than causal, and alternative models were tested to evaluate competing explanations.

Accordingly, the present study aims to examine whether problematic social media use and doomscrolling sequentially account for the association between post-earthquake traumatic stress and limited future time perspective. Based on the theoretical framework outlined above, the following hypotheses were proposed:

- H1. Post-earthquake traumatic stress positively predicts problematic social media use.
- H2. Problematic social media use positively predicts doomscrolling behavior.
- H3. Doomscrolling negatively predicts future time perspective (focus on limitations).
- H4. Post-earthquake traumatic stress positively predicts limited future time perspective.
- H5. Problematic social media use mediates the relationship between post-earthquake traumatic stress and doomscrolling.
- H6. Doomscrolling mediates the relationship between problematic social media use and future time perspective.
- H7. Problematic social media use and doomscrolling sequentially account for the relationship between post-earthquake traumatic stress and future time perspective.

## Method

### Participants and procedure

A total of 414 undergraduate students from a public university in Kahramanmaraş, Türkiye, participated in this study. Kahramanmaraş was among the provinces most directly affected by the February 6, 2023 earthquakes, making this context theoretically relevant for examining post-earthquake traumatic stress and its associations with digital behavior and future time perspective. Participants were recruited using convenience sampling. The sample consisted of 198 females (47.8%), 207 males (50.0%), and 9 individuals (2.2%) who preferred not to disclose their gender. Students from different academic years were represented in the sample; however, detailed information on variables such as income and employment status was not collected in sufficient detail to be included in the analyses.

Data were collected in classroom settings under the supervision of a faculty member from the Department of Psychological Counseling and Guidance. Participation was voluntary, and informed consent was obtained prior to data collection. Questionnaires were completed in approximately 10–15 min.

Although participants were recruited from a disaster-affected region, the present study did not assess specific indicators of objective earthquake exposure (e.g., injury, bereavement, or material loss). Instead, the study focused on self-reported traumatic stress symptoms as an indicator of individuals’ subjective psychological responses within a post-earthquake context. This approach is consistent with prior research suggesting that psychological responses to large-scale disasters may not correspond directly to objective exposure and can be meaningfully captured through subjective stress-related experiences. Accordingly, the construct of post-earthquake traumatic stress in the present study should be interpreted as reflecting perceived psychological impact rather than verified exposure to specific disaster-related events.

The study protocol was approved by the Ethics Committee of Istanbul Medipol University, Social Sciences Institute (Approval No: 2025/274).

### Measures

#### Post-earthquake traumatic stress

Post-earthquake traumatic stress was assessed using the Post-Earthquake Trauma Scale developed by Tanhan and Kayri.[33] The scale consists of 20 items rated on a 5-point Likert scale, with higher total scores indicating higher levels of post-earthquake traumatic stress. In the present study, the scale demonstrated excellent internal consistency (Cronbach’s α = 0.92). No reverse-coded items were included in the scoring of this measure, and higher scores indicate higher levels of the construct.

#### Problematic social media use

Problematic social media use was measured using the Social Media Disorder Scale developed by Van den Eijnden et al. [12] and adapted into Turkish by Erinç et al. [34]. The scale comprises 9 items assessing problematic patterns of social media use. Higher total scores indicate higher levels of problematic social media use. The Turkish adaptation study provided evidence for the validity and reliability of the measure. [34] In the current sample, the scale showed high internal consistency (Cronbach’s α = 0.90). No reverse-coded items were included in the scoring of this measure, and higher scores indicate higher levels of the construct.

#### Doomscrolling

Doomscrolling behavior was assessed using the Doomscrolling Scale developed by Satici et al. [14]. The scale includes 15 items rated on a 7-point Likert scale, with higher scores reflecting greater tendencies toward compulsive exposure to negative online content. Prior research has supported the validity and reliability of this scale [14].

#### Future time perspective

Future time perspective was measured using the Future Time Perspective Scale [35], adapted into Turkish by Soylu and Özekes [36] The scale consists of two subdimensions: focus on opportunities and focus on limitations. Items are rated such that higher scores on each subdimension reflect stronger endorsement of that temporal orientation. Evidence for the validity and reliability of the Turkish form was reported in the adaptation study [36]. In the present study, internal consistency coefficients were satisfactory (α = 0.90 for opportunities; α = 0.87 for limitations). No reverse-coded items were included in the scoring of this measure, and higher scores on each subdimension indicate stronger endorsement of that temporal orientation.

#### Data analysis

All analyses were conducted using R [37]. Prior to hypothesis testing, the data were screened for missing values and outliers. Normality assumptions were evaluated using skewness and kurtosis statistics, with all values falling within the acceptable range of − 2 to + 2, indicating no substantial deviation from normality.

Confirmatory factor analyses (CFA) were conducted using the lavaan package [38]. Internal consistency of the scales was assessed using Cronbach’s alpha coefficients.

To test the proposed indirect effects model, path analysis with observed composite variables was performed, which is conceptually equivalent to PROCESS Model 6 [39]. Given the cross-sectional nature of the data, the model was specified as an indirect effects model rather than a causal mediation model. No covariates were included in the primary model in order to preserve model parsimony and because the study was designed to examine the hypothesized relationships among the focal constructs. Nevertheless, demographic variables such as age may be relevant to digital behavior and future time perspective and should be considered in future research.

In the model, post-earthquake traumatic stress was specified as the independent variable, problematic social media use as the first mediator, doomscrolling as the second mediator, and future time perspective (focus on limitations) as the dependent variable. Variance inflation factor (VIF) values ranged between 1.84 and 2.27, indicating no multicollinearity concerns.

Indirect effects were estimated using bootstrapping with 5,000 resamples. Bias-corrected 95% confidence intervals were used to determine statistical significance. An indirect effect was considered statistically significant if the confidence interval did not include zero.

To further evaluate model robustness, two alternative models were tested. The first specified problematic social media use as a single mediator between post-earthquake traumatic stress and limited future time perspective, whereas the second included doomscrolling as the sole mediator. Model fit was evaluated using χ², CFI, TLI, RMSEA, and SRMR indices. Additionally, Akaike Information Criterion (AIC) and Bayesian Information Criterion (BIC) values were used to assess model parsimony and comparative fit.

## Results

Descriptive statistics of the study variables are presented in Table 1. The mean score for post-earthquake traumatic stress was 58.72 (SD = 20.90). The mean scores for problematic social media use and doomscrolling were 29.48 (SD = 15.54) and 15.68 (SD = 6.82), respectively. For future time perspective, the mean scores were 26.30 (SD = 9.59) for focus on opportunities and 12.22 (SD = 4.82) for focus on limitations. Skewness and kurtosis values indicated no substantial deviation from normality.

**Table 1:** Descriptive statistics of study variables

Variable	*N*	Mean	SD
Post-Earthquake Traumatic Stress	414	58.72	20.90
Problematic Social Media Use	414	29.48	15.54
Doomscrolling	414	15.68	6.82
Future Time Perspective (Opportunities)	414	26.30	9.59
Future Time Perspective (Limitations)	414	12.22	4.82

Pearson correlation coefficients among the study variables are presented in Table 2. Post-earthquake traumatic stress was positively associated with problematic social media use, doomscrolling, and focus on limitations, and negatively associated with focus on opportunities. Problematic social media use showed a strong positive correlation with doomscrolling and was positively related to focus on limitations. Doomscrolling was also positively associated with focus on limitations and negatively associated with focus on opportunities. Overall, the correlations were in the expected directions and were consistent with the proposed model.

**Table 2:** Pearson correlations among study variables

Variables	1	2	3	4	5
1. Post-Earthquake Traumatic Stress	1				
2. Problematic Social Media Use	0.657^**^	1			
3. Doomscrolling	0.650^**^	0.583^**^	1		
4. Future Time Perspective (Opportunities)	-0.468^**^	-0.383^**^	-0.419^**^	1	
5. Future Time Perspective (Limitations)	0.605^**^	0.616^**^	0.573^**^	-0.320^**^	1

**Note. p < .01

The direct and indirect effects of the proposed indirect effects model are presented in Table 3.

**Table 3:** Direct and indirect effects of the proposed indirect effects model

Effect Type	Path	B	Boot SE	β	t	p	95% CI LL	95% CI UL
Direct Effects	Post-Earthquake Traumatic Stress → Problematic Social Media Use (A1)	0.488	0.026	0.656	18.377	< .001	0.434	0.538
	Problematic Social Media Use → Doomscrolling (A2)	0.275	0.022	0.627	12.138	< .001	0.230	0.318
	Post-Earthquake Traumatic Stress → Doomscrolling (A3)	0.077	0.016	0.237	4.575	< .001	0.045	0.111
	Doomscrolling → Limited Future Time Perspective (B1)	0.085	0.049	0.120	1.711	.086	-0.012	0.184
	Post-Earthquake Traumatic Stress → Limited Future Time Perspective (C′)	0.074	0.012	0.323	6.156	< .001	0.050	0.097
	Problematic Social Media Use → Limited Future Time Perspective (B2)	0.095	0.023	0.309	4.147	< .001	0.051	0.141
Indirect Effects	Post-Earthquake Traumatic Stress → Problematic Social Media Use →	0.011	0.006	0.049	1.736	.082	-0.001	0.024
	Doomscrolling → Limited FTP	0.046	0.011	0.203	4.139	< .001	0.025	0.069
	Post-Earthquake Traumatic Stress → Problematic Social Media Use → Limited FTP	0.006	0.004	0.028	1.518	.128	-0.000	0.016
	Total Indirect Effect	0.064	0.009	0.281	6.773	< .001	0.046	0.084
Total Effect	Post-Earthquake Traumatic Stress → Limited Future Time Perspective (c)	0.139	0.009	0.604	14.890	< .001	0.120	0.157

**Fig. 1:**
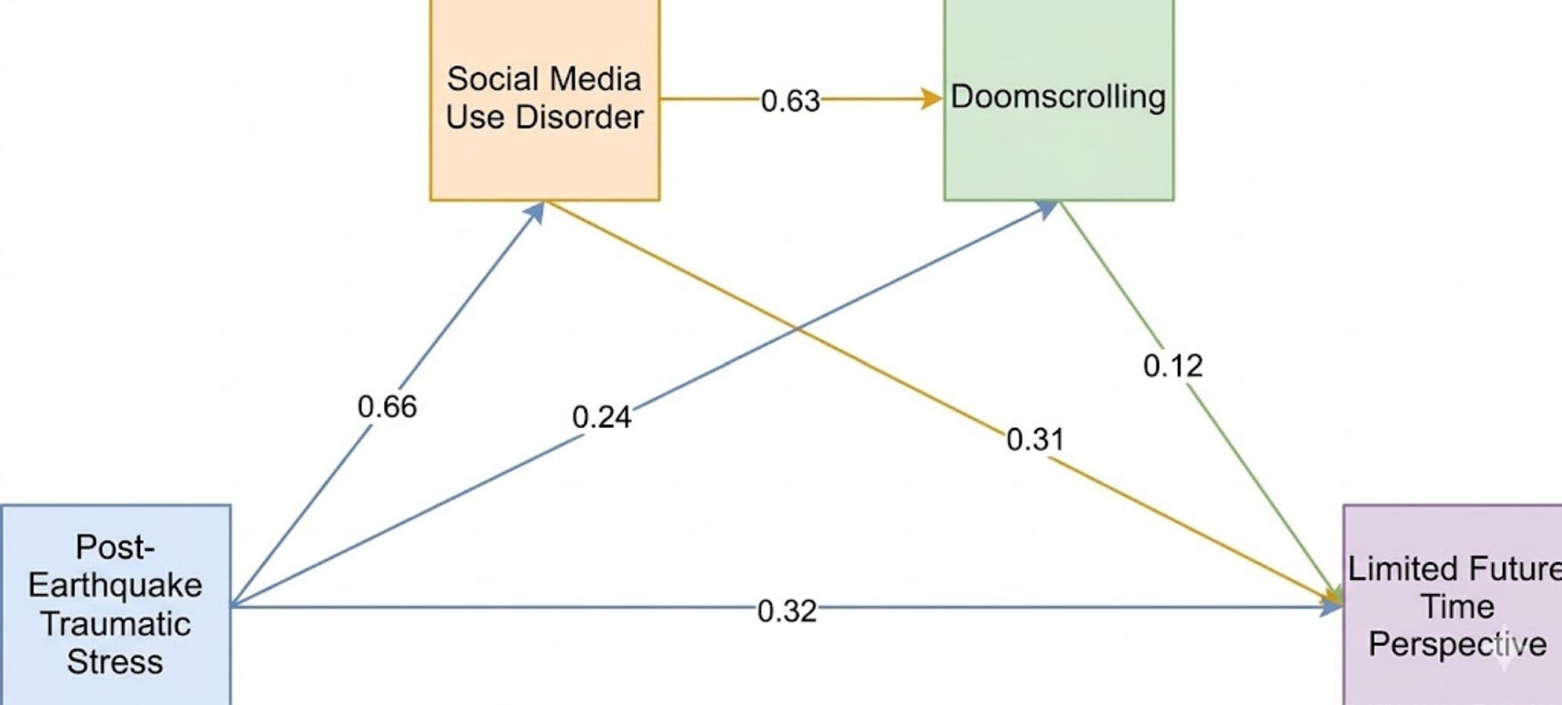
Proposed indirect effects model with standardized path coefficients

The proposed indirect effects model examined the effects of post-earthquake traumatic stress on limited future time perspective through problematic social media use and doomscrolling (see Fig. 1).

Post-earthquake traumatic stress significantly predicted problematic social media use (β = 0.656, *p* < .001) and doomscrolling (β = 0.237, *p* < .001). Problematic social media use also significantly predicted doomscrolling (β = 0.627, *p* < .001).

With respect to the outcome variable, post-earthquake traumatic stress (β = 0.323, *p* < .001) and problematic social media use (β = 0.309, *p* < .001) were significant predictors of limited future time perspective. However, doomscrolling did not significantly predict the outcome (β = 0.120, *p* = .086).

Bootstrapping analyses indicated that the indirect effect of post-earthquake traumatic stress on limited future time perspective through problematic social media use was significant (95% CI [0.025, 0.069]). In contrast, the indirect effect through doomscrolling alone was not significant, and the full sequential indirect pathway (post-earthquake traumatic stress → problematic social media use → doomscrolling → limited future time perspective) did not reach statistical significance (95% CI [− 0.001, 0.024]). The total indirect effect was significant (95% CI [0.046, 0.084]), and the total effect of post-earthquake traumatic stress on limited future time perspective was strong and statistically significant (β = 0.604, *p* < .001). The standardized path coefficients of the model are presented in Fig. 1.

All reported paths include standardized coefficients; significance levels are presented in Table 3.

As shown in Table 4 above, the proposed indirect effects model and the proposed single-mediator model with problematic social media use fit the data acceptably. However, due to the very low degrees of freedom (df = 1), global fit indices such as the root mean square error of approximation (RMSEA) should be interpreted cautiously since even the smallest changes could imply significant misspecification. the model with doomscrolling as the sole mediator showed a poorer fit compared to the single-mediator model including problematic social media use, as shown by the higher values for RMSEA and information criterion values.

**Table 4:** Competing model analyses

Model	χ² (df)	CFI	TLI	RMSEA	SRMR	AIC	BIC
Indirect effects model	4.12 (1)	0.989	0.948	0.082	0.032	8452.31	8491.20
PSMU-only model	2.76 (1)	0.993	0.971	0.064	0.021	8448.90	8480.75
Doomscrolling-only model	6.35 (1)	0.975	0.902	0.112	0.047	8463.54	8495.38

Given the very low degrees of freedom (df = 1) in the tested models, RMSEA values should be interpreted cautiously, as this index may be sensitive and potentially inflated in small-df models

χ² chi-square statistic, *CFI*  Comparative Fit Index, *TLI*  Tucker–Lewis Index, *RMSEA*  Root Mean Square Error of Approximation, *SRMR*  Standardized Root Mean Square Residual, *AIC*  Akaike Information Criterion, *BIC*  Bayesian Information Criterion

Although the proposed single-mediator model had slightly better values for the Akaike information criterion (AIC) and Bayesian information criterion (BIC) than the proposed indirect effects model, the difference was very small.

To further evaluate the plausibility of the proposed model, additional models were tested (see Table 4). The results indicated that both the proposed indirect effects model and the single-mediator model including problematic social media use demonstrated acceptable model fit. It should be noted that the tested models have very few degrees of freedom (df = 1). In this case, the results of the global fit indices, especially the RMSEA, should be treated with caution, where very small variations may not necessarily indicate problems with the model. On the other hand, the model that only includes doomscrolling as the mediator had a poor fit to the data, reflected by the increased RMSEA and information indices. The single-mediator model was slightly more parsimonious than the proposed model; however, the difference was marginal. In light of the theoretical relevance of the proposed model, the indirect effects model was retained for further interpretation.

## Discussion

Overall, the findings of the present study indicate that post-earthquake traumatic stress is associated not only with individuals’ perceptions of the future but also with patterns of digital engagement. Consistent with prior research, traumatic stress symptoms appear to be linked to a more limited future time perspective, characterized by reduced expectations and a diminished sense of control [40–44]. From a theoretical standpoint, this pattern aligns with existing work suggesting that large-scale stressors may disrupt temporal orientation and future-directed thinking [41, 45].

Importantly, the findings further suggest that digital behaviors may represent a meaningful component of this broader adjustment process, rather than merely reflecting secondary outcomes. Given the cross-sectional nature of the data, these associations should be interpreted as non-causal. Nevertheless, the observed pattern is consistent with theoretical perspectives emphasizing the impact of large-scale stressors on both cognitive representations of the future and everyday behavioral tendencies.

Beyond this general relationship, the findings highlight the role of problematic social media use as a key behavioral pathway linking post-earthquake traumatic stress to a more limited future time perspective. This result is consistent with prior research suggesting that increased engagement with digital platforms during periods of uncertainty may reflect attempts to regulate distress and restore a sense of control [46–49]. At the same time, such engagement may evolve into more persistent and less controllable patterns of use, which have been associated with elevated psychological distress and difficulties in self-regulation [50, 51].

Rather than reflecting mere frequency of use, problematic social media use may represent a broader behavioral tendency characterized by diminished control over digital engagement. In this sense, it may function as a mechanism through which trauma-related distress becomes linked to more enduring cognitive patterns, including how individuals perceive and structure their future.

In contrast, although doomscrolling was significantly associated with both post-earthquake traumatic stress and problematic social media use, it did not emerge as a significant predictor of future time perspective within the full model. This finding suggests that doomscrolling may reflect a more immediate and situational response to perceived threat, characterized by repetitive exposure to negative information and short-term increases in distress [17, 18, 23].

While such behavior may intensify emotional responses in the short term, it may not be sufficient on its own to influence more stable cognitive constructs such as future time perspective. Accordingly, doomscrolling may be better understood as a context-dependent behavioral response, rather than a primary mechanism shaping future-oriented cognition.

Another important consideration concerns the conceptual and empirical overlap between problematic social media use and doomscrolling. Although these constructs share common features related to excessive engagement and reduced behavioral control, they differ in scope and specificity. Problematic social media use reflects a broader, generalized pattern of dysregulated engagement across digital contexts, whereas doomscrolling represents a more specific, content-driven behavior characterized by repeated exposure to negative information.

In the present study, the attenuation of the effect of doomscrolling after accounting for problematic social media use may suggest that variance associated with doomscrolling is partially subsumed under this broader behavioral tendency. At the same time, the distinct pattern of associations observed supports the conceptual distinction between these constructs. Taken together, these findings indicate that while related, problematic social media use and doomscrolling operate at different levels of digital behavior and should not be treated as interchangeable.

Importantly, the findings also contribute to ongoing discussions regarding the functional role of digital behaviors in contexts of uncertainty. From the perspective of Uncertainty Reduction Theory, information seeking is typically understood as an adaptive response to ambiguity and threat [30–33]. However, the present findings suggest that when such behaviors become excessive or dysregulated, they may no longer serve a purely adaptive function. Instead, repeated engagement with digital platforms may reflect a shift from short-term coping toward more persistent maladaptive patterns, which may be associated with less adaptive future-oriented cognition.

In this sense, the findings extend existing theoretical perspectives by highlighting that digital behaviors may simultaneously function as both coping mechanisms and potential sources of psychological strain, depending on their intensity and regulation.

Taken together, the findings suggest that digital behaviors following traumatic stress are not uniform but may operate at multiple levels. While some behaviors, such as doomscrolling, may reflect immediate and context-dependent responses to uncertainty, others, such as problematic social media use, may represent more stable behavioral patterns with broader implications for cognitive and emotional functioning.

Accordingly, the relationship between post-earthquake traumatic stress and future time perspective may be better understood as a multi-layered process involving both situational and more enduring forms of digital engagement. This perspective highlights the importance of distinguishing between different types of digital behavior when examining psychological adjustment in the aftermath of large-scale stressors.

At a more applied level, these findings suggest that examining patterns of digital engagement may provide additional insight into how individuals adapt to traumatic stress experiences, particularly in university populations. Rather than focusing exclusively on symptom reduction, it may also be informative to consider how individuals interact with digital environments and whether these patterns are associated with variations in psychological adjustment. Although the present study does not directly evaluate intervention strategies, the findings highlight the potential relevance of digital behavior as an important contextual factor in understanding responses to traumatic stress.

## Limitations

This study has several limitations that should be considered when interpreting the findings. First, the cross-sectional design precludes any causal conclusions regarding the relationships among post-earthquake traumatic stress, digital behaviors, and future time perspective. Although the proposed model was theoretically grounded, the findings should be interpreted as reflecting associations within an indirect effects framework rather than directional or causal pathways. Future research employing longitudinal or experimental designs is needed to clarify temporal ordering and potential causal mechanisms.

Second, although participants were recruited from a region directly affected by the February 6, 2023 earthquakes, the study did not assess specific indicators of earthquake-related exposure (e.g., personal injury, loss of relatives, displacement, or material damage). Accordingly, the findings reflect self-reported traumatic stress symptoms rather than objectively verified exposure to disaster-related events. Future studies would benefit from incorporating both objective and subjective indicators of disaster exposure to better capture the complexity of trauma-related experiences.

Third, all variables were assessed using self-report measures, which may introduce biases such as social desirability and common method variance. Although the measures demonstrated good internal consistency, reliance on self-report data limits the extent to which the findings can be generalized beyond subjective experiences.

Finally, the sample consisted of undergraduate students from a single institution, which may limit the generalizability of the findings to other age groups, cultural contexts, or populations with varying levels of disaster-related exposure. Future research should consider more diverse and representative samples to enhance the external validity of the proposed model.

## Conclusion

This study examined the relationship between post-earthquake traumatic stress and future time perspective, with a particular focus on the role of digital behaviors. The findings indicate that post-earthquake traumatic stress is associated with both problematic social media use and a more limited future time perspective. Notably, problematic social media use emerged as a key factor accounting for this relationship, whereas doomscrolling did not independently predict future time perspective.

These results suggest that digital engagement following traumatic stress may reflect more than increased time spent online; rather, it may represent a broader pattern of dysregulated behavior linked to how individuals cognitively orient toward the future. The findings further indicate that not all forms of digital behavior operate in the same way, highlighting the importance of distinguishing between general patterns of use and more specific content-driven behaviors.

Overall, this study contributes to the literature by integrating post-earthquake traumatic stress, digital behavior, and future time perspective within a unified framework. Although the full indirect pathway was not supported, the results consistently underscore the role of problematic social media use as a central component of digital behavioral processes in post-disaster contexts.

## Data Availability

The datasets generated and/or analyzed during the current study are not publicly available due to ethical and privacy considerations but are available from the corresponding author on reasonable request.

## Abbreviations

FTP: Future time perspective
PSMU: Problematic social media use
URT: Uncertainty Reduction Theory
CFA: Confirmatory factor analysis
VIF: Variance inflation factor
